# Delayed discharge in inpatient psychiatric care: a systematic review

**DOI:** 10.1186/s13033-024-00635-9

**Published:** 2024-04-06

**Authors:** Ashley-Louise Teale, Ceri Morgan, Tom A. Jenkins, Pamela Jacobsen

**Affiliations:** Department of Psychology, University of Bath, Bath, BA2 7AY UK

**Keywords:** Delayed discharge, Bed blocking, Delayed transfer, Psychiatric inpatient, Inpatient treatment, Prolonged stays, Length of stay

## Abstract

**Background:**

Delayed discharge is problematic. It is financially costly and can create barriers to delivering best patient care, by preventing return to usual functioning and delaying admissions of others in need. This systematic review aimed to collate existing evidence on delayed discharge in psychiatric inpatient settings and to develop understanding of factors and outcomes of delays in these services.

**Methods:**

A search of relevant literature published between 2002 and 2022 was conducted on Pubmed, PsycInfo and Embase. Studies of any design, which published data on delayed discharge from psychiatric inpatient care in high income countries were included. Studies examining child and adolescent, general medical or forensic settings were excluded. A narrative synthesis method was utilised. Quality of research was appraised using the Mixed Methods Appraisal Tool (MMAT).

**Results:**

Eighteen studies from England, Canada, Australia, Ireland, and Norway met the inclusion criteria. Six main reasons for delayed discharge were identified: (1) accommodation needs, (2) challenges securing community or rehabilitation support, (3) funding difficulties, (4) family/carer factors, (5) forensic considerations and (6) person being out of area. Some demographic and clinical factors were also found to relate to delays, such as having a diagnosis of schizophrenia or other psychotic disorder, cognitive impairment, and increased service input prior to admission. Being unemployed and socially isolated were also linked to delays. Only one study commented on consequences of delays for patients, finding they experienced feelings of lack of choice and control. Four studies examined consequences on services, identifying high financial costs.

**Conclusion:**

Overall, the findings suggest there are multiple interlinked factors relevant in delayed discharge that should be considered in practice and policy. Suggestions for future research are discussed, including investigating delayed discharge in other high-income countries, examining delayed discharge from child and forensic psychiatric settings, and exploring consequences of delays on patients and staff. We suggest that future research be consistent in terms used to define delayed discharge, to enhance the clarity of the evidence base.

**Review registration number on PROSPERO:**

292515.

**Date of registration:**

9th December 2021.

**Supplementary Information:**

The online version contains supplementary material available at 10.1186/s13033-024-00635-9.

## Background

Delayed discharge, also termed ‘bed blocking’ and ‘delayed transfer of care,’ refers to when patients remain in hospital beyond the time they are determined to be clinically fit to leave [1, 2]. It is an international challenge, costly to individuals, health services and governments [3, 4], impacting physical health settings, and also psychiatric inpatient services [5].

Psychiatric inpatient stays are one of the most expensive forms of treatment for mental health conditions, particularly when compared to care delivered in community settings [6]. Prolonged stays in mental health hospitals likely increase resource use and as such financial expenditure. This is particularly concerning in instances of delayed discharge when stays are determined to not be of clinical benefit. Delayed discharge also could prevent admission of new patients, contributing to bed crises, where there are not enough beds for all who require admission [7]. This can have consequences on the course of recovery for newly referred patients, either delaying admission, contributing to inappropriate placements, or leading to individuals being placed out of area [7, 8]. Extended hospital stay could also detrimentally impact the delayed patient themselves, preventing their return to usual day-to-day functioning and make returning to the community increasingly difficult [9, 10].

Existing reviews have examined predictors of longer stays in psychiatric inpatient settings, finding substance use and being employed are associated with shorter length of stay; while being female, having a diagnoses of mood or psychotic disorders and use of Electroconvulsive Therapy are associated with longer stay [11]. However, there is not to our knowledge a systematic review collating evidence examining delayed discharge in psychiatric settings. As delayed discharge is a unique experience, distinct from long stay driven by clinical need, it requires separate focus to further understand this specific experience.

Furthermore, a large body of evidence has examined delayed discharge in physical health settings with several systematic reviews, examining causes and outcomes. Such reviews have found that delayed discharges were linked to problems in discharge planning, transfer of care difficulties and patient age [12, 13]. Outcomes for services included overcrowding and financial costs, whereas outcomes for patients included infections, depression, reduction in activities and mortality. There may be both overlapping and non-overlapping factors associated with delayed discharge between physical and psychiatric inpatient settings. For example, inpatient psychiatric services may differ in organisational structure, daily workings, and treatment focus from general medical services. The clinical population might also differ in psychiatric and physical health settings, for example in age, socio-economic status, and other demographic, plus clinical factors. As such, it is vital that separate attention be given to the area of psychiatric care.

This systematic review aims to fill the current research gap and synthesise existing literature on psychiatric delayed discharges. We aimed to synthesise the available international data from high-income countries, as the prevalence and underlying reasons for delayed discharge are likely to be highly sensitive to context and heterogeneous across countries. This is due to factors such as different models of healthcare funding, and the varying social role of the family in providing care, for example. Developing in-depth understanding of the causes and consequences of delays in a psychiatric inpatient context is important in informing practice and policies at a service, organisational, societal, and government level. This could help develop ways to reduce occurrence of delays and mitigate any negative impacts.

The aim of this review was to increase understanding of what is known about factors influencing delayed discharge in adult psychiatric inpatient settings. Secondary aims were to examine outcomes of delayed discharge for patients and compare findings across different psychiatric settings and age groups.

## Method

The systematic review protocol was pre-registered on PROSPERO before the review was started and the searches were run (PROSPERO: 292515). The review is reported in line with PRISMA guidelines [14, 15]. The primary research question of this review is: What is known about factors associated with delayed discharge from inpatient psychiatric care settings?

Secondary research questions were:

1. What are the outcomes for those who have experienced delayed discharge from inpatient psychiatric settings, for example, in mental health outcomes, health outcomes, readmissions and quality of life?
2. What are the outcomes on services in terms of resources and costs from delayed psychiatric inpatient discharge?
3. What are the experiences of staff and patients of delayed discharge from inpatient mental health wards?
4. Are there differences between types of inpatient services, including acute, rehabilitation or specialist inpatient wards, in factors and costs?
5. Are there differences between working age adults and older adults, in experiences of delayed discharge?

### Search Strategy

Initial searches were conducted on the 15th of January 2022, and updated on the 5th of August 2022. Pubmed, PsycInfo and Embase were searched.

Search terms (Appendix B in supplementary materials) were developed through examining key words of published studies on the topic, reviewing the terms used in comparative reviews based in physical health settings and thesaurus mapping. Terms included: “delayed discharge,” “bed blocking” and “long stays.” Search terms were piloted on each database prior to running the final search.

The search included studies published from 2002. A 20-year search timeframe was selected, as psychiatric inpatient care has adapted in response to changing need and updated knowledge over time. As such, studies published before 2002 are likely to be less relevant to current practice.

Following database searches, reference lists of included papers were examined, to identify any relevant studies missed in the search. A forward citation search was also conducted, to identify any relevant studies that were cited in the included papers.

### Inclusion and exclusion criteria

Studies were included if they reported data related to delayed discharge or associated outcomes, in adult psychiatric inpatient wards. Specialist and rehabilitation psychiatric inpatient settings were included. Studies of any design were included, providing they were published in a peer-reviewed journal. Both quantitative and qualitative studies were included.

Studies exploring delayed discharge in child or adolescent units and/or forensic units were excluded. This was because the causes and outcomes of delays in such settings are likely unique, given the specialist context. For example, there is likely different systemic involvement from families and different governing legislation in these contexts. As such, it was determined that such settings were too disparate, and synthesising studies from these settings together with adult psychiatric settings could lead to inaccurate conclusions. Physical health settings were also excluded, given the different processes, procedures and treatment focus involved in such settings. In addition, reviews have already been conducted examining delayed discharge from such settings. Studies not conducted in high-income countries were also excluded. In this review, we included high-income countries as defined by World Bank criteria, accessed in January 2022 [16] (see Appendix C in supplementary material for the list of included countries). Globally, countries differ in the conceptualisation of mental health and provisions offered, therefore, limiting this review to only high-income countries would enable comparisons to be made.

### Study selection and data extraction

Screening was conducted using Covidence Systematic Review Software [17]. All records were independently double-screened by two reviewers at both title/abstract and full-text stage. Conflicts were resolved by discussion to reach consensus, with referral to the senior author (PJ) when needed.

A standardised template was used for data extraction, with all included studies being independently double extracted by two reviewers, with consensus achieved by discussion where needed.

### Analysis

A narrative synthesis method was used. For data examining reasons for delayed discharge, a deductive approach was taken initially. Authors identified possible reasons for delays based on existing literature and organised data under these categories/themes. Any data that did not fit into the pre-defined categories was pooled as ‘other’. All categories were then reviewed, with particular attention placed on the ‘other’ categories, to determine if additional categories need to be added or existing categories adapted. Sub-categories were identified when appropriate through coding. Once categories were established, the number of papers which reported each reason/factor were tabulated and data was reviewed to examine relationships, exploring both links and disparities within and between studies. The final synthesis was checked by three authors (AT, TJ, and CM), to achieve final agreement.

Data relating to outcomes/consequences of delayed discharge was synthesised in a similar way, with data initially organised into three categories: (1) consequences for patients, (2) consequences for service, (3) consequences for staff. Categories were reviewed by the authors following synthesis. Financial costs were converted to US dollars by the authors to support comparison.

Quality assessment of the included studies formed part of the synthesis with the appraisal of quality considered in the interpretation of results.

### Quality Assessment

Quality assessment of studies was completed during the synthesis stage. In the protocol, we initially outlined that the Quality Assessment Tool for Studies of Diverse Designs (QATSDD) would be utilised [18]. However, following a trial of this tool with the included papers, we noted disparities in interpretations between authors. Therefore, the Mixed Methods Appraisal Tool (MMAT) was established to be a more suitable appraisal of quality for the included studies. The MMAT was developed for assessing and comparing the quality of studies using quantitative, qualitative and mixed-methods design, in one tool [19]. This tool was selected as studies of different designs were included in the review and this tool allows for quality appraisal across five different study types, distinguishing between methodology.

Two initial screening questions were answered to determine appropriateness of using the MMAT to assess quality of the study (are there clear research questions and do the collected data address the research questions). If screening questions are not passed, this tool is deemed inappropriate. Providing the screening questions were passed, quality was assessed on five questions within one of five categories. The category in which questions were answered was determined by study design. The MMAT discourages from scoring and assigning qualitative labels to describe quality, instead advises a more detailed evaluation of quality [19]. This approach has therefore been taken in this paper.

To achieve reliable and accurate quality ratings, every study was quality rated by two members of the research team and conflicts were discussed to reach consensus.

## Results

### Identification of studies

**Fig. 1:**
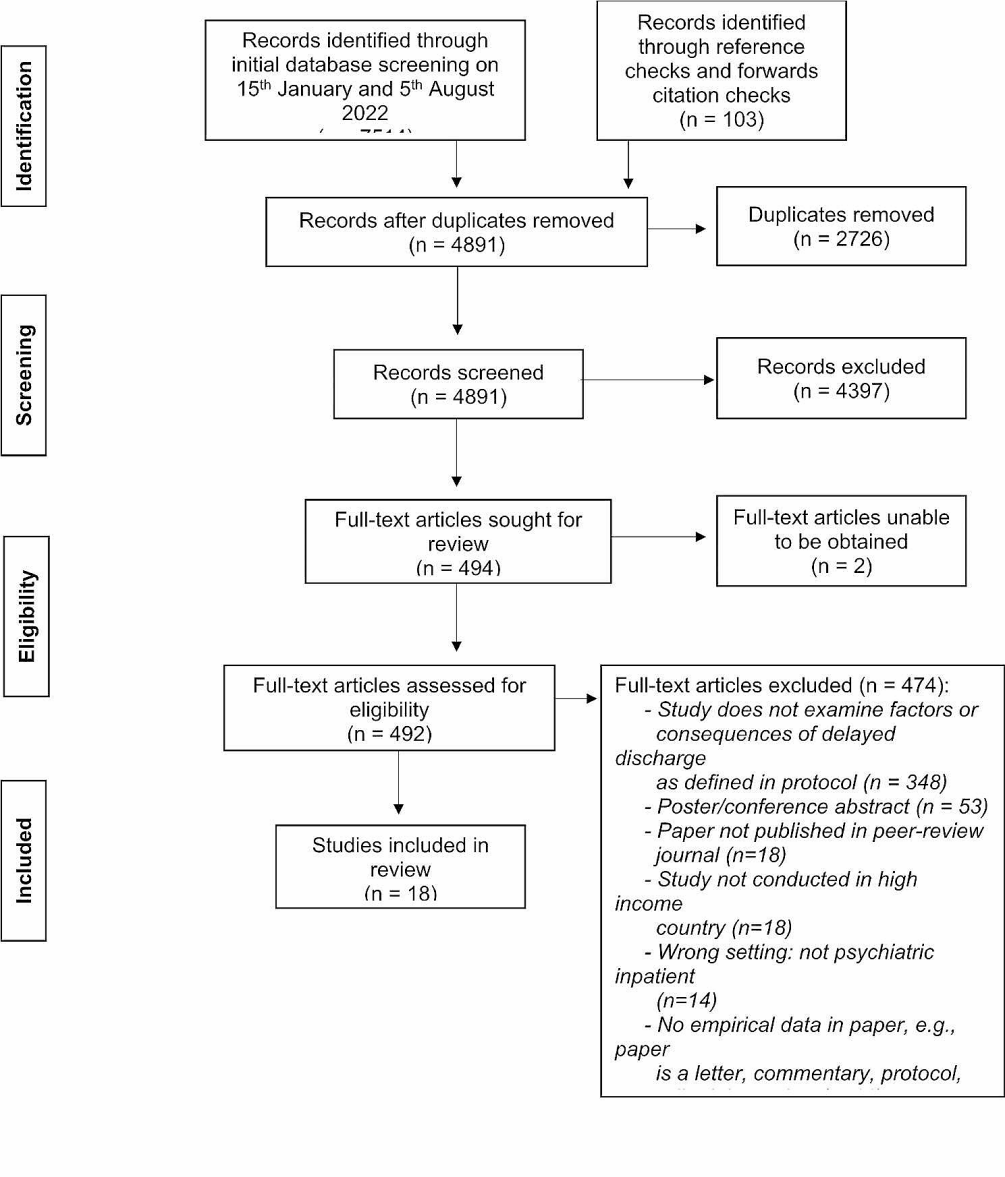
Preferred reporting items for systematic reviews and meta-analyses (PRISMA) flowchart

Figure 1 (PRISMA flowchart) shows the study selection process. After removing duplicates, a total of 4891 papers were identified for screening. 4397 papers were excluded at title and abstract stage. Full texts were then obtained for 492 papers. Two full texts could not be obtained via the library service and the authors did not respond to a request for the paper. There were four papers obtained that were erratum’s, all of which related to excluded studies that were not examining delayed discharge and as such, were not linked to the included studies. Following full text screening 18 papers were eligible for inclusion. Each paper represented a different study.

### Study characteristics

Table 1 shows the characteristics of the 18 included studies. Twelve of these studies examined delayed discharge as a primary outcome, with three of these studies specifically examining Housing Related Delayed Discharge (HRDD). HRDD is defined as instances where delayed discharge is attributed to housing issues. The remaining studies (*n* = 6) reported delayed discharge as secondary outcomes. Fifteen studies were of quantitative observational design, two studies used mixed methodologies and one was qualitative.

**Table 1:** Overview of studies included in the review: table organised by country, setting and year

First author (year); country	Primary Aim of Study	Study Design; Data type	Setting Type	Sample (n; sample type; age)	Data Collection Method	Main findings
Onyon (2006); England [32]	Delayed discharges	Observational Audit; Quantitative	PICU	80 patients/88 discharge records; discharges in one year; WAA (19–61)	Retrospective audit of patient notes	Delays significantly associated with schizophrenia diagnosis (*p* = 0.03) and being admitted from other inpatient units or the community compared to from forensic settings (*p* = 0003) Black ethnicity was significantly associated with prolonged delay in discharge (*p* = 0.032)
Haw (2017); England [34]	Out of area admissions	Observational Audit; Quantitative	PICU	170 admissions/168 patients; admissions over one year; age not reported	Audit using data collection forms completed or checked by patient’s consultant psychiatrist	Wait for bed to become available, delay in identifying appropriate placement, funding dispute between trusts, delay in home area assessing patient, communication difficulties between staff and home area team, delay finding suitable placement, delay in assessment from staff from a specialist placement, patient not known to services, patient turned down by placements, and delay by Ministry of Justice. Delays significantly associated with schizophrenia diagnosis (*p* < 0.05) and discharge to acute ward (*p* < 0.0001)
Tyrer (2006); England [31]	Measuring Bed Inventory	Observational Audit; Quantitative	Acute	740 patients occupying 668 beds; admissions over one year; age not reported	Bed requirement inventory	No significant association with gender, age, ethnicity, marital status, or form of admission. However, those admitted from hostels or housing charity (50%) had highest levels of delayed discharge (*p* = 0.048)
Commander; England [36]	Long stay	Observational Audit; Quantitative	Acute	38 patients; all long-stay patients identified on census days; WAA (16+)	Census data, nurse reports and psychiatrist/nurse responses on Community Placement Questionnaire	Delays linked to accommodation to go to, awaiting adaptations to home, accepted for place but lacked funding, awaiting forensic assessment
Impey (2013); England [33]	Delayed Discharges	Observational Audit; Quantitative	Acute	65 patients; all beds in service; WAA (younger than 70)	Survey completed by inpatient team and computerised records check	Delays linked with awaiting accommodation, or awaiting transfer to other hospital More likely to be women, higher mean age.
Cowman (2016); Ireland [27]	In-patients housing needs	Observational Audit; Quantitative	Acute	Not reported; inpatients over 12 months	Information derived from nurses	Delays linked to accommodation needs, awaiting nursing home placement
Lewis (2006); England [28]	Delayed discharges	Mixed-methods	Mental Health trusts	35 trusts; NHS trusts at one time-point; WAA and OA	Mixed methods survey	Delays linked to patient/family exercising choice, funding, awaiting assessment, further NHS Care, Domiciliary package, residential, nursing home
Paton (2004); England [37]	LOS and delayed discharges	Observational Audit; Quantitative	OA	91 occupied beds/65 patients; all beds in samples services; OA (63+)	Interview with consultant, discussion at eligibility panel, The Camberwell Assessment of Need for the Elderly (CANE); The Neuropsychiatric Inventory (NPI); The Abbreviated Bristol Activities of Daily Living Scale.	Delays linked to no alternative placement available, lack of money to finance placement, relatives refused to fund placement, relative/patient turned placement down, insufficient specialist staff resources for placement or returning home.
Hanif (2008); England [20]	Delayed discharges	Observational Audit; Quantitative	OA	50 patients; people discharged over 3 months; OA (61+)	Review of medical records	Delays linked to unavailable destination placement; carer delay, awaiting nursing/residential home assessment and feedback, funding issue; setup of homecare, delays in patient transfer to destination; patient out of area
Tucker (2017); England [30]	LOS and delayed discharges	Observational Audit; Quantitative	OA	216 admissions; patients admitted over 6 months, OA (65+)	Ward round staff reports, nursing staff collected discharge data.	Delays linked to difficulties finding suitable care home; waiting suitable care home vacancy; difficulties accessing funding for care home; waiting assessment by care home; difficulties arranging appropriate and timely community care. Significant predictors included greater cognitive impairment, being in fair-excellent health, seeing social care prior to admission.
Poole (2014); England [35]	Delayed discharges	Observational Audit; Quantitative	Acute, PICUs, and OA	237 inpatient beds; all in patients over three months; WAA and OA	Audit of electronic records and staff questionnaire	Delays linked to no bed available, no suitable placement found, awaiting funding decision, some patients had no right to funding, care package to support person at home not in place, waiting bed in secure facility, assessment for placement underway. Half of delayed discharge group were female, length of stay significantly longer in younger delayed adults (*p* = 0.014), black Caribbean patients over-represented in delayed discharges; only one patient in younger delayed discharge sample was employed.
Chuah (2022); Australia [26]	HRDD	Qualitative	Acute	10; HRDD; WAA	Qualitative interviews	Outcome of HRDD included lack of choice and control which reduced mental well-being; decreased physical health and created a more difficult anticipated transition back into the community. Two participants also described benefits of staying in hospital, e.g., finding it preferable to the alternative of being homeless.
Honey (2022); Australia [24]	HRDD	Observational Audit; Quantitative	Acute	55 HRDD and 55 non-HRDD; WAA (15–64)	Medical record review	NDIS administrative delay and rejection from rehabilitation services impacted HRDD group only. Significant predictors of HRDD: Difficulty identifying appropriate community support services, not being employed at admission, and not having a history of criminal justice system involvement. Other significant associations with HRDD were a diagnosis of schizophrenia or other psychotic disorders, physical disability/health condition, aggressive or violent behaviour, NDIS status on discharge, housing stability, legal status during admission, self-harm.
Nguyen (2022); Australia [25]	HRDD	Mixed-methods	Acute	59 patients; people with HRDD over one year; WAA (15–64) + 8 staff for interviews	Medical record review and qualitative interviews with staff	Reasons for HRDD: Patient does not want to return to previous accommodation, difficulty finding accommodation, lack of housing options, lack of clear and effective pathways to find and access accommodation, awaiting property repairs or resolution of social conflicts to return to accommodation, social housing related delays, application for supported/social housing rejected, difficulty finding community support packages, request for support services rejected, lack of support network, family conflict, family does not want the patient to return to live with them, application for NDIS rejected, delays relating to funding of NDIS. Some characteristics common in HRDD sample were being male, not being employed, being unmarried, diagnosis of schizophrenia, history of violent/aggressive behaviour, drug and alcohol use. Staff also noted participation restrictions/high support needs, ongoing symptoms, and lack of insight higher in delayed group. HRDD cost $4,054,149 in 2018.
Aflalo (2015); Canada [23]	Prolonged hospital stay	Observational Audit; Quantitative	Acute	262 admissions; admissions with 30 days + over one year; WAA and OA (18+)	Medical record review and data collection from care team	Delays linked to difficulty finding or lack of available resources for support/placement, appropriate resources lacking or difficult to find, no longer acute but ongoing assessment to determine appropriate resources, administrative and (or) social issues (for example, waiting for a court date or waiting transfer to jail), ongoing family discussion, ongoing liaison process with community care staff, waiting for specific treatment. Most patients had a diagnosis of mental and behavioural disorders, factors influencing health status and contact with health services. Schizophrenia most prevalence mental health diagnosis.
Little (2015); Canada [21]	Delayed discharge	Observational Audit; Quantitative	Acute	68 hospitals; WAA and OA (18+)	Clinical Assessment data from Resident Assessment Instrument Mental health	Significant associations with being delayed included: male gender, older age, speaking foreign language, being homeless, receiving more days of contact from almost every profession the week preceding admission, being unmarried, schizophrenia diagnosis and cognitive disorders. The financial cost of caring for an ALC patient is roughly $7650.
Little (2019); Canada [22]	Delayed discharge	Observational Audit; Quantitative	Acute	76 and 184 patients; inpatient admissions over two years; WAA and OA	Clinical Assessment data from Resident Assessment Instrument Mental health and Wait Time Information System	Variables associated with higher odds of delay were impairment in activities of daily living, moderate to severe cognitive impairment, no insight into mental health, disorders of childhood/adolescence, intellectual disabilities, impairment in Activities of Daily Living, aggressive behaviours, history of substance abuse, having six or more previous admissions, being middle and older age, male, speaking a primary language other than English or French, being visited less often by a social relation, social isolation and not being married. Clinical variables that had lower odds of 30 + day delay were psychosis/schizophrenia, severe symptoms related to social withdrawal, and moderate-to-severe symptoms of depression.
Berg (2005); Norway [29]	Bed occupancy	Observational Audit; Quantitative	Acute	23 patients; identified on a random day; WAA (22–56)	Method of data collection unclear	Delays linked to waiting for secondary resident treatment. In the delayed group there were more men, mean age was lower, all had a psychotic illness. Delays accounted for 54.8% of cost of treatment.

Note. Abbreviations: HRDD, Housing related delayed discharge; DD, delayed discharge; OA, Older adult; WAA, Working age adult; PICU, Psychiatric Intensive Care Unit; ALC, Alternate Level of Care; NHS, National Health Service; NDIS, National Disability Insurance Scheme

In the included studies, there was a range of psychiatric inpatient settings: psychiatric/general mental health units (*n* = 11), Psychiatric Intensive Care Units (PICUs) (*n* = 2), older adult psychiatric units (*n* = 3) and Mental Health Trusts (*n* = 1). One study looked across three inpatient settings: acute psychiatric, PICU and older adult. Studies were conducted in five high income countries (England = 10, Ireland = 1, Australia = 3, Canada = 3, and Norway = 1). There were no studies from any other high-income countries, identified in the search.

### Quality Assessment

The MMAT quality scores are shown (Table 2). One included study [20] did not meet initial criteria to be assessed using this tool, as the research questions were unclear.

All studies were of fairly good quality, with all studies meeting at least three out of five of the quality assessment criteria. Quality was highest in Australian and Canadian studies, with included papers in these countries meeting all five quality assessment criteria [21–26]. Quality assessment ratings indicate that three quantitative descriptive studies included, did not clearly report use of a representative sample or appropriate measures. Ratings per question are shown in Table two.

**Table 2:** M-MAT quality assessment ratings

Study	Criteria					Total number of criteria met (/5)
*Qualitative Study*	**1.1**	**1.2**	**1.3**	**1.4**	**1.5**	
Chuah	✓	✓	✓	✓	✓	5
*Quantitative Descriptive Studies*	**4.1**	**4.2**	**4.3**	**4.4**	**4.5**	
Impey	✓	✓	✓	✓	✓	5
Honey	✓	✓	✓	✓	✓	5
Onyon	✓	✓	✓	X	✓	4
Haw	✓	✓	✓	✓	✓	5
Little 2015	✓	✓	✓	✓	✓	5
Little 2019	✓	✓	✓	✓	✓	5
Berg	✓	?	?	✓	✓	3
Tyrer	?	?	✓	✓	✓	3
Tucker	✓	✓	?	✓	✓	4
Poole	✓	✓	✓	✓	✓	5
Paton	✓	?	✓	✓	✓	4
Cowman	✓	✓	X	✓	?	3
Commander	✓	✓	✓	✓	✓	5
Afilalo	✓	✓	✓	✓	✓	5
*Mixed Methods Study*	**5.1**	**5.2**	**5.3**	**5.4**	**5.5**	
Nguyen	✓	✓	✓	✓	✓	5
Lewis & Glasby	✓	?	✓	✓	?	3

Note. Denotation of “X” indicates criteria was not met, “✓” indicates criteria was met, “?” unclear if criteria met. Quality criteria and questions included in Appendix C

### Research Q1

#### What is known about factors associated with delayed discharge?

Thirteen studies identified reasons for delayed discharge (Table 1). The results showed that there are many complex reasons for delays with often overlapping contributing factors. We categorised reasons for delay into six categories: (1) accommodation needs, (2) difficulty securing rehabilitation or community support, (3) finance/funding challenges, (4) family/carer factors, (5) forensic factors, (6) patient being out of area.

The most common reason for delays was due to accommodation and placement factors. This was identified as a contributing reason for delay in twelve studies and a further two studies assessed Housing-Related Delayed Discharge (HRDD), suggesting accommodation factors contributing to delay in these cases. Accommodation/placement factors included limited availability of placements (*n* = 7), difficulty finding appropriate placements (*n* = 5), awaiting or undergoing placement assessment (*n* = 3), challenges in person returning to accommodation (*n* = 3), e.g., awaiting repairs or adaptations to their home, individuals being rejected from placement (*n* = 2), patients/family rejecting placement (*n* = 2) and awaiting transfer (*n* = 1). It should be noted that one of the studies which examined specific accommodation factors was unable to be quality assessed due to not having clear research questions and therefore did not meet the screening criteria for assessment with the MMAT [20], and two studies only met three of the five quality assessment criteria, with queries regarding the quality of measures used and analysis technique for one study [27], and some difficulties integrating and meeting the full quality criteria for the mixed methods approaches used in the second [28]. The second reason identified for delays was difficulty sourcing support for the person to enable discharge, such as community, rehabilitation, and homecare support. This contributed to delays in twelve studies. Eight of these studies met four to five of the quality assessment criteria, one was not able to be assessed [20], and three only met three of the five quality assessment criteria [27–29]. A third reason for delay was finance/funding challenges identified in nine studies. These included challenges obtaining funding, patients/families’ refusal to pay for placements and funding applications being rejected. Six studies identified family/carers factors in creating delays, such as family conflict, family not wanting the person to live with them and ongoing family discussion. The quality of two of the studies identifying family and finance factors should be considered, as one of these studies was unable to be quality assessed due to a lack of clear research questions [20] and a second met only three of the five quality assessment criteria [28]. The fifth reason identified in this review as contributing to delay was forensic factors, which accounted for delays in three studies, all of good methodological quality. Forensic delays incorporated delay by Ministry of Justice and awaiting forensic assessment. Person being out of area was highlighted as a reason for delay in only one study and it was not possible to quality assess this study due to no specific research questions identified [20], suggesting limited exploration or evidence for out of areas contributing to delays.

Fourteen studies included in this review examined the demographic and clinical factors relevant in delays, with eight conducting significance testing to establish associations. Significant associations with delay were having a diagnosis of schizophrenia or other psychotic disorder (*n* = 4), cognitive impairment (*n* = 3) and type/amount of service input prior to admission (*n* = 3). All studies reporting these significant results were of a good methodological quality, achieving at least four of the five MMAT quality criteria. Results were mainly consistent across those studies which examined significance, however, there was one study of good quality that did not find significant association with schizophrenia diagnosis [22]. The impact of physical health differed between Australia and England, where in one English study having fair-excellent health was more associated with delays [30], though two Australian studies found poorer physical health linked to delays [24, 25]. Findings related to demographic characteristics, including gender, age, ethnicity, socio-economic status, were inconsistent across studies. The only consistent finding was that a smaller proportion of the delayed group were employed (*n* = 3). One of these studies found significant association between being unemployed and delayed discharge. The two other studies found only one member of the delayed group was employed, less than non-delayed groups, though this was not significance tested. There was some indication that being not being married and lacking a support network, was higher in delayed groups. One study found significant relationships to being unmarried and another finding that the delayed group were visited significantly less often by relatives. The other studies did not conduct significance testing. However, there was no significant relationship related to marriage between delayed and non-delayed groups in two studies [22, 31]. One of these studies only clearly met three of the quality assessment criteria [31], though the other met all five quality assessment criteria. Being male was significantly associated with delays in two Canadian studies [21, 22]. No significant association with gender was found in other studies.

The supplementary materials provide additional analysis of results for research question one, further describing each study’s findings. Additional materials also include tables showing tabulation of which study examined each variable.

### Research Q2

#### What are the outcomes for those who have experienced delayed discharge from inpatient psychiatric settings for example, in mental health outcomes, health outcomes, readmissions and quality of life?

Only one study examined individual outcomes of delayed discharge for patients [26]. As such, there is limited data to draw conclusions to answer this research question. The study that evaluated patient outcomes was of qualitative design and good quality. The study explored Housing-Related Delayed Discharge (HRDD) in Australia for 10 patients using semi-structured interviews. They found consequences of lack of choice and control for patients, which impacted mental wellbeing, physical health and created a sense of anticipation for transition to community. Some participants highlighted a positive outcome of delayed discharge in preventing homelessness.

### Research Q3

#### What is the outcome on services in terms of resources and costs from delayed psychiatric inpatient discharge?

Four studies assessed financial costs of delayed discharge for services, providing limited evidence in terms of financial outcomes. Each study focused on a different country. At an old age psychiatry unit in England, delayed discharges were estimated to cost over $855,820 for the year [20]. Notably, this study was not quality assessed due to the omission of research questions. In a high-quality paper from Australia, HRDD cost the health district $2,828,174 over one year [25]. While both papers present yearly costs, there is disparity in area covered, contributing to difficulty making comparisons regarding financial expenditure. Two studies calculated financial expenditure and did not present the cost per year. In a Canadian study, using the median number of delayed days (*M* = 17), it was calculated that the average cost incurred by one episode of delayed days was approximately $5,746 [21]. Furthermore, in Norway, $491,406 was allocated to delays on the acute ward included in the study, though methodological quality might be queried, due to lack of clarity on whether the sample was representative and the appropriateness of measures utilised [29]. The information necessary to calculate costs per year or costs per delayed day, to enable comparisons to be made across studies, has not included in the studies.

Aside from financial costs, no other type of outcome for services were assessed.

### Research Q4

#### What are the experiences of staff and patients of delayed discharge from inpatient mental health wards?

None of the included studies explored specific experiences of delayed discharge for staff. Some information on experiences for patients is detailed in question two.

### Research Q5

#### Are there differences between types of inpatient services, including acute, rehabilitation or specialist inpatient wards, in factors and costs?

This systematic review identified studies in acute psychiatric, older adult and Psychiatric Intensive Care Unit (PICU) settings. Only one study included Learning Disability inpatient care settings [28]. This study was of mixed-method design and met three quality assessment criteria. No studies reported data from rehabilitation units. There were few differences identified between types of setting. Prevalence of delayed discharge was highest in older adult settings (56.9%) [30] and PICU settings (51.1%) [32], compared to working age adult settings (18–32%) [31, 33]. However, the highest proportion of delayed days was found in acute psychiatric settings in Norway acute psychiatric units (54.8%) [29]. More information on prevalence is provided in supplementary materials.

Reasons for delay did not vary much across type of setting. There is a potential service difference in the impact of physical health in delays, as having fair-excellent health was more associated with delays in an English older adult study [30], while in a working age adult sample in Australian studies [24, 25], having poor health was more associated with delays. However, this could represent a disparity in country. There were some other differences across countries found. Forensic reasons for delay were only found in the UK (*n* = 2), as was due to patient being out of area (*n* = 1). In UK settings, there was no significant difference found in gender between those delayed and those not [30, 34], though there was in Canada [21]. England and Australia were the only countries identifying funding issues as contributing to delay. Each country will have its own respective funding system, which could impact delays. For example, two Australian studies identified difficulties with their own National Disability Insurance Scheme [24, 25].

### Research Q6

#### Are there differences between working age adults and older adults, in experiences of delayed discharge?

Only five of the included studies looked specifically at older adult settings, all of which were in the UK. A further five studies, from the UK and Canada, included older adults within their sample, despite not examining a specific older adult setting.

The highest proportion of inpatients experiencing delayed discharge were from older adult settings, with one study identifying 56.9% [30] of inpatients experiencing delays. There were lower rates of delayed patients in working age adult psychiatric inpatient settings in comparison, with 3.5% [21, 25] to 39.1% [29] of patients experiencing delay. Similarly, two studies in Canada identified that a higher proportion of older adults made up the delayed group compared to the non-delayed group, suggesting that older adult inpatients are more likely to experience delay [21, 22]. However, two English studies found delayed discharge was not associated with age [31, 35]. One of these studies met only three quality assessment criteria, with lack of clarity regarding the quality of sampling and representativeness of the sample [31].

In terms of reasons for delay, no clear differences were found across age groups. Although when limiting comparisons to studies conducted in the UK, family/carer factors was identified as a reason for delay more frequently in older adult samples (*n* = 3) compared to studies looking at working age adults (*n* = 1). To support this finding, one study in England found that eight older adult trusts identified patient/carer exercising choice as a reason for delay, whereas the same was true for only four working age adult trusts [28]. However, this finding cannot be generalised across all countries. There is also some indication that cognitive impairment/dementia might increase likelihood of delay in older adult samples, as two studies identified the role of dementia and greater cognitive impairment in the delayed older adult groups [20, 30]. A further two studies examined the impact of cognitive impairment, finding association with delay [21, 22]. However, these studies included working age samples, so it is unclear who in the sample this impacted. In addition, physical health status could cause delays differently in older adult populations. In an older adult UK sample having fair-excellent health was more associated with delays [30], whereas two Australian studies in working age adult inpatient settings found poorer physical health increased delays [24, 25]. This difference could however be attributed to country or setting. Funding was identified as a reason for delay in all studies in older adult settings (*n* = 5), but the same was not true for the other setting types. Forensic factors were not found to be a reason for delay in any of the studies with older adult inpatients, conversely patient being out of area was only identified as a reason for delay in an older adult sample [20].

## Discussion

This systematic review aimed to fill a research gap and examine factors contributing to delayed discharge in adult psychiatric inpatient settings and explore associated consequences. This adds a unique contribution to the evidence base, which predominantly has focused on delayed discharge from physical health settings. Eighteen studies were included for synthesis.

The findings suggest that there are varying inter-related reasons for delay, including accommodation or placement needs, difficulties securing the required support services, funding and finance challenges, family/carer factors, forensic factors and the person being out of area. There were mixed findings regarding demographic and clinical characteristics associated with delays. However, this review showed that delays could be associated with the person having diagnosis of schizophrenia or other psychotic disorder, cognitive impairment, being unemployed and receiving increased service input prior to admission.

There were only a few studies that commented on outcomes of delays. Only one study examined outcomes for patients, identifying feelings of lack of choice and control, while four studies looked at financial outcomes for services, finding large costs associated with delays. This points to a lack of evidence examining the outcomes and experiences of psychiatric delayed discharge, and therefore requires further attention in research.

This review adds to and expands on existing findings, identifying similarities and differences between longer stay generally. For example, one review [11] found that long stay was associated with mood and psychotic disorders, use of Electroconvulsive Therapy, and being female. Being married, employed, and using substances were associated with a shorter stay [11]. Our review found that psychiatric delayed discharge was also associated with diagnosis of schizophrenia or other psychotic disorder and being unemployed. However, we found delayed discharge to be associated with cognitive impairment and increased service input prior to admission, but not gender or treatment. This could suggest some important differences in those at risk of delays or those requiring longer inpatient treatment. It is important to note however, the review by Gopalakrishina and colleagues did not distinguish between those patients with long stay clinically warranted and delayed discharge patients [11]. It would be of benefit for future research on long stay patients to better define their sample based on those who clinically needed treatment or longer stay patients in the context of delayed discharge, allowing similarities and differences to be better explored. This will support policy makers and service managers to better identify those at risk of delays that are not clinically necessary, and those who might need additional clinical input. The findings in this review provide some suggestion that there could be benefit in considering a person’s social context when they are admitted to psychiatric inpatient care, including their living situation at admission, employment status and cognitive functioning. Identifying patients at higher risk of delays earlier in admission might be useful, to ensure more time be given to organise and find appropriate accommodations, placements and service support and facilitate discharge. Wider policy and structural changes are needed, such as improving the availability of appropriate accommodation placements.

It is important to highlight that there were discrepancies across studies in language used to term delayed discharge, e.g., ‘alternate level of care,’ ‘waiting days’ and ‘prolonged stay.’ Due to such discrepancies in definitions and terminology, during the screening process it was at times difficult to determine if studies were focused on delayed discharge or longer lengths of stay clinically required. In this review, studies were excluded if the focus was unclear to prevent incorrect conclusions being drawn related to the unique experience of delayed discharge. However, this means other relevant findings might have been missed. It would therefore be useful for future research on psychiatric inpatient care to ensure clarity in the terminology and definitions used in reports. There were also discrepancies in the way financial costs related to delays were reported, i.e., whether reported as cost per day, cost per year. This made comparing the costs across countries challenging and prevented clear conclusions being drawn. Future research should therefore aim to ensure clarity when reporting financial expenditure, for example, by calculating the daily cost of delays. It is important to highlight that only eighteen studies were identified over the 20-year search period, suggesting this area has not yet been subject to much research focus. All high-income countries met inclusion, but the final sample included studies from only five countries. It might have been expected that studies in other high-income countries be identified, particularly given the expensive nature of inpatient stays and as such delayed discharge. It might be beneficial for future research to further examine delayed discharge in psychiatric settings across other countries, particularly in the USA and EU. For the purposes of this review, studies not conducted in high-income countries were excluded. This was because lower-income countries might experience different factors contributing to delays due to differences in healthcare funding and social factors. As such, separate attention should be given to these settings, to understand similarities or differences in reasons for delays across low- and mid- income countries. Studies on forensic psychiatric settings and child and adolescent settings were also excluded in this instance, so again, there might be benefit in future research examining these areas.

Furthermore, future research could look not only at factors creating delays, but those causing longer delays. Some of the studies in this review began examining this, but more research in this area could be of interest. Finally, while the quality of included studies was relatively high, the studies were primarily of quantitative audit design and infrequently conducted significance testing. As such, further exploration of associations using significance testing would strengthen the evidence base.

## Conclusion

In conclusion, 18 studies identified reasons for delayed discharge, including accommodation and placement related factors, challenges securing appropriate support, funding difficulties, family/carer factors, forensic factors and person being out of area. Delay was associated with having a diagnosis of schizophrenia or other psychotic disorder, cognitive impairment, increased service involvement prior to admission, and being unemployed. Service, societal and policy changes might be indicated, to improve accommodation and care provisions following discharge. Future research should continue to examine prolonged inpatient psychiatric stays, ensuring to distinguish between long stays and delayed discharge and improve clarity in terminology used.

### Electronic supplementary material

Below is the link to the electronic supplementary material.

Supplementary Material 1

## Data Availability

The data on which this review is based will be made publicly available on publication. A link to data for anonymous peer-review is here: https://osf.io/j4kng/?view_only=1fbf2558d9d044bbb1778fccd5fd6f51.
