# Supplementary material for: Delayed discharge in inpatient psychiatric care: a systematic review
Source: Int J Ment Health Syst. 2024 Apr 6;18:14. doi: 10.1186/s13033-024-00635-9 (PMC10998409; doi:10.1186/s13033-024-00635-9)
Supplement: Supplementary file 1 — Supplementary Material 1 [file 13033_2024_635_MOESM1_ESM.docx]

**Delayed discharge in inpatient psychiatric care: A Systematic Review
Journal:** International Journal of Mental Health Systems

Ashley-Louise Teale^*^, Ceri Morgan, Tom A. Jenkins & Pamela Jacobsen

Dr Ashley-Louise Teale*****, University of Bath, Department of Psychology, Taunton and Somerset Foundation Trust, Bath, UK (ORCID ID: 0000-0002-1756-7711)

Dr Ceri Morgan, University of Bath, Department of Psychology, Taunton and Somerset Foundation Trust, Bath, UK (ORCID ID: 0000-0002-2417-8677).

Tom A. Jenkins, University of Bath, Department of Psychology, Bath (ORCID-ID: 0000-0001-7875-4417).

Dr Pamela Jacobsen, University of Bath, Department of Psychology, Bath, UK (ORCID ID: 0000-0001-8847-7775).

*** Corresponding author:** [alvt20@bath.ac.uk](mailto:alvt20@bath.ac.uk)

**Supplementary Material.**

**Appendix A: Additional Synthesis**

These supplementary materials provide additional analysis and further exploration of the factors associated with delayed discharge, to provide a more detailed answer to the first research question. At the end of this document, the prevalence of delayed discharge has also been further discussed.

***Research Question 1:
What is known about factors associated with delayed discharge?***

At the synthesis stage, it was observed that the included studies made conclusions about both the reasons contributing to delay and the clinical/demographic variables associated with delay. Results were presented following this two-part format in the main article, and the same structure will be applied here.

REASONS FOR DELAY

Thirteen of the included studies identified reasons relevant in delayed discharge. Through data extraction and synthesis, reasons for delayed discharge were organised into six categories. Table 1 depicts the studies which identified each factor as a reason for delays.

1. Accommodation/placement needs

Twelve studies identified accommodation and placement needs as an important factor in contributing to delayed discharge. This factor captures challenges in securing the discharge location in terms of availability, suitability, and patient acceptance, sometimes conceptualised as the person as having nowhere else to go. Accommodation/placement needs were often found to be the most common implicating factor in delays. For example, one study highlighted how 98% of delayed patients had accommodation needs [1], with another noting that having nowhere to go contributed to 75% of delays [2]. In addition, the three studies in Australia, all focused specifically on Housing Related Delayed Discharge (HRDD), putting added emphasis on this being a cause for concern in delays. The category of accommodation/placement needs is broad, and as such has been further dissected into six sub-categories:

*1.1. Limited availability of placements:* Limited availability of housing and placements was another factor highlighted as causing delay, identified as a cause in seven studies. Six of these studies were in the UK, across various settings. Specifically, it was found that unavailable placement caused delay in 12/27 delayed patients in one older adult setting [3] and 20% in another [4]. One study commented that waiting for a care home vacancy contributed to delays in an older adult psychiatric unit, though did not quantitatively calculate to what extent [5]. In one study examining multiple inpatient settings, 31.6% of delays were due to no bed being available and 7.6% due to waiting for a bed in secure facilities [6]. It was highlighted that in some cases, following the wait, placement in secure inpatient units was no longer needed [6]. In a PICU setting, waiting for a bed to be available contributed to delays in 38 out of 51 delayed patients [7]. Waiting for a nursing home placement was also identified as a contributor to delays in Ireland, impacting 6% of the sample [1]. These findings suggest this reason for delay could be more relevant to a range of settings in the UK, though to varying extents. One additional Australian study commented on limited availability of rehabilitation facilities contributing to delays, though specific quantitative evidence for this is difficult to unpick [8].

*1.2. Difficulty finding appropriate accommodation/nowhere to go:* Five studies highlighted delays as being caused by challenges finding appropriate accommodation or placements. This accounted for 23% of delayed discharges in one study in England [6] and 92% of Housing Related Delayed Discharge (HRDD) in an Australian study [8]. A further study in Canadian services identified that difficulty finding appropriate long-term care placement contributed to delays, but the amount of the sample impacted by this was unclear [9]. One study in older adult inpatient services noted that difficulty finding suitable care home was a reason for delay but provided no quantitative data to suggest the extent of this in causing delays [5]. The final study identifying difficulty finding appropriate accommodation as a reason for delay was in a Psychiatric Intensive Care Unit (PICU) setting, and this reason was identified as contributing to delay in six out of the 51 delayed patients [7]. As such, difficulty finding placement or accommodation, seems to impact delays across country and type of inpatient setting. A further two studies found that patients were delayed due to having no accommodation to go to [2] and no alternative to inpatient accommodation [4].

*1.3. Awaiting assessment:* Awaiting an assessment by home teams or placement location also contributed to delays in three studies. In one study examining a range of settings, 6.4% of delayed patients were due to assessment for placement taking place [6]. Two studies in older adult services also commented on awaiting assessment at care home or nursing/residential home as causing delay [3, 5]. While this represents a range of service types, all the studies were in England, suggesting this wait for assessment might be unique to this location.

*1.4. Difficulties returning to previous accommodation:* Three studies, two English and one Australian, noted delays caused by difficulty returning patients to their previous accommodation. In the English studies, it was found that one patient was delayed due to awaiting adaptations to be made to their home [2] and in another study it was identified that delays were caused as some individuals had homes that were not suitable to return too, though no figures were given indicating how many in the sample this impacted [4]. As mentioned in the above sub-category, but also relevant here, one Australian study found that 31% of people were delayed as they did not want to return to their previous accommodation and some patients were delayed due to awaiting repairs to their home and resolution of conflicts [8].

*1.5.* *Placement rejections:* Two studies identified that delays were due to patients being rejected from placements/accommodations. One study in PICU highlighted how delay for one patient was caused by being turned down by multiple placements [7]. An Australian study highlighted that 7% and 10% of the sample was rejected for social and supported housing [8]. This study further contributed that discrimination prevented patients accessing housing, but also general challenges completing applications to secure housing [8]. There is also some evidence of patients/carers rejecting housing options (n=2). This was identified as relevant to one patient in one English study [4], however in an Australian study, 31% of delayed cases were due to patient’s not wanting to return home [8]. One further study noted reason for delay as patient/family exercising choice in 15 National Health Service (NHS) trusts, which perhaps could be relevant to declining placements [10].

*1.6. Awaiting transfer:* Another reason for delays were patients awaiting transfer to new placement (n=1). This is conceptualised separately from above, as in these instances the reason for wait is unclear and it could be due to factors other than awaiting bed availability. In one study it was found that delays in transfer impacted two patients [3]. However, it is unclear if this was delay in transfer to a different hospital facility, or accommodation placement.

*1.7. Process challenges:* It is of value to note that one study in Australia commented on staff difficulties navigating the process of applying and organising accommodation [8]. This study also highlighted difficulties with individuals being able to access the internet to find accommodation and having leave to view placements [8].

*2. Difficulty Sourcing Community/Rehabilitation Support*

Twelve studies identified difficulty sourcing rehabilitation or community support contributed to delayed discharge. Seven of these studies were in England and one Ireland. All English older adult psychiatric units noted this as contributing to delays (n=3). In one of these samples, it was found that three of the 17 delayed patients were delayed due to challenges setting up homecare [3], though in another study 30% of delays were due to insufficient specialist staff resources for next accommodation [4]. The third study in older adult services qualitatively reported that most delays in patients returning home were due to difficulties arranging appropriate and timely care packages [5]. Two studies in acute inpatient settings found that waiting for nursing home placements [1] and waiting transfer to another hospital [11] was a reason for delay. One study examining multiple types of inpatient settings identified that 7.6% of patients were delayed due to not having in place an element of a care package to support them at home [6]. Furthermore, in a PICU setting, delays in the home area assessing patients contributed to delayed discharge in six cases [7]. One final study in England, examining NHS trusts, found that difficulties identifying appropriate specialist services for older people in the community contributed to delays, and waiting for further care packages contributed to delay across all age groups [10].

Four studies across other countries also noted difficulties with obtaining community or rehabilitation support. This included two Australian samples, whereby in one sample, 37% of HRDD was due to difficulty finding community support packages, 12% due to rejection from rehabilitation services and 10% due to rejection from community support services [8]. The second study similarly found that difficulty identifying appropriate community support services significantly predicted delayed discharge [12]. One Canadian study identified that 12% of delays were due to ongoing liaison processes with community care staff and 6% of delayed patients were waiting for a specific treatment [9]. The final study identifying this factor as causing delays was in Norway and noted that 47.8% of delays was due to wait for secondary residential treatment [13].

*3. Finances/funding*

Funding challenges were identified as a reason for delayed discharge in nine studies, seven of which were based in England. All the studies in English older adult inpatient settings highlighted funding challenges as a reason for delay (n=3). In one such study four out of 27 delays were attributed to funding issues [3], and in a second, 20% of delays were due to lack of money to finance placements or because relatives refused to pay [4]. The third older adult study qualitatively noted challenges accessing funding for care home placements as contributing to delays [5]. In a PICU setting it was found that funding difficulties contributed to three out of 51 of the patient delays [7]. Funding challenges also was found to be a reason for delays in two studies looking across settings, with 13 mental health trusts highlighting this reason for delay, with particular emphasis noted on challenges finding funding for older adult specialist services [10] and 10.1% of delayed discharges across settings found to be due to awaiting funding decisions [6]. One study in acute settings found that five patients were accepted for a placement but lacked funding [2]. It was also highlighted in one study how there were a group of people who had no right to funding, such as failed asylum seekers, and this contributed to discharge delays [6]. These results show potential funding issues for accommodations, placements and support services in England contribute to delays. The final two studies which highlighted funding as contributing to delayed discharge, examined HRDD in Australia [8, 12]. These studies specifically noted challenges with their National Disability Insurance Scheme (NDIS), finding applications for NDIS were rejected or patients awaited decisions from NDIS. High rent was also qualitatively attributed as a reason for delays in an Australian study [8].

*4. Family/Carer*

Six studies identified family/carer factors that contributed to delayed discharge, three of these based in England. Carer delay accounted for 117 days delay and impacted 12 out of 27 patients in one older adult inpatient sample [3]. However, in another older adult sample family/carer factors were only relevant in one out of 30 delayed patients, whereby a relative and patient turned a down a discharge placement [4]. Similarly, patient/family exercising choice was a reason for delay in eight older adult inpatients, four adult inpatients and three learning disability inpatients [10]. Two Australian studies noted family/carer factors leading to HRDD. One study highlighted several family/carer reasons for delays including lack of support (48%), family conflict (38%) and family not wanting patient to live with them (31%) [8]. However, in a sample of 55 people with HRDD, ongoing family conflict was the only noted family/carer factor, though impacting 26 delayed patients [12]. The final study commenting on family/carer factors was based in Canada, finding that ongoing family discussion was relevant in delay in 8% of delayed cases [9].

*5. Forensic Delays*

Three studies identified forensic reasons for delays, though findings suggest this is relevant in a small number of cases. One study in England noted that in two delayed discharge cases, there was a wait for a forensic assessment [2]. A second study set in England found that one patient was delayed due to awaiting Ministry of Justice to approve a transfer [7]. The third study was conducted in Canada and found that forensic administrative issues contributed to delay in 30% of delayed patients, this included patients awaiting court dates or transfer to jail, which prompted delays for 30% of patients [9]. It is, however, unclear if this figure accounted for other types of administrative delays, or forensic administrative delays specifically. Nonetheless, forensic reasons might impact a higher proportion of patients in Canada.

*6. Patient Out of Area*

Only one study identified patient being out of area as contributing to delays [3]. Despite this being relevant in only one study and in only one case within the sample, this caused a 201-day delay. As such, it was determined that this should be highlighted within this review.

*Final Note.*

As discussed in the main article, but worth highlighting again, there seems to be a range of reasons for delay. The subject matter is complex, with many overlapping reasons for delay noted. Often, there is no one singular reason for a patient.

**Table 1** Table depicting which studies identified which factor as relevant in delayed discharge.

|  | **Housing needs** | **Accommodation needs sub-categories** | | | | | | | **Difficulty securing rehabilitation or community support** | **Finance / funding challenges** | **Family / carer factors** | **Forensic** | **Patient out of area** |
| --- | --- | --- | --- | --- | --- | --- | --- | --- | --- | --- | --- | --- | --- |
|  |  | **Difficulty finding placement** | **Limited placement availability** | **Awaiting transfer to placement** | **Awaiting assessment** | **Rejection from placements** | **Patient / family rejected placement** | **Challenges returning to placement** |  |  |  |  |  |
| Onyon (2006) |  |  |  |  |  |  |  |  |  |  |  |  |  |
| Haw (2017) | X | X | X |  |  | X |  |  | X | X |  | X |  |
| Tyrer (2006) |  |  |  |  |  |  |  |  |  |  |  |  |  |
| Commander (2016) | X |  |  |  |  |  |  | X |  | X |  | X |  |
| Impey (2013) | X |  |  |  |  |  |  |  | X |  |  |  |  |
| Cowman (2016) | X |  | X |  |  |  |  |  | X |  |  |  |  |
| Lewis (2006) | X |  |  |  |  |  | X |  | X | X | X |  |  |
| Paton (2004) | X |  | X |  |  |  | X | X | X | X | X |  |  |
| Hanif (2008) | X |  | X | X | X |  |  |  | X | X | X |  | X |
| Tucker (2017) | X | X | X |  | X |  |  |  | X | X |  |  |  |
| Poole (2014) | X | X | X |  | X |  |  |  | X | X |  |  |  |
| Chuah (2022) | HRDD |  |  |  |  |  |  |  |  |  |  |  |  |
| Honey (2022) | HRDD |  |  |  |  |  |  |  | X | X | X |  |  |
| Nguyen (2022) | X | X | X |  |  | X |  | X | X | X | X |  |  |
| Aflalo (2015) | X | X |  |  |  |  |  |  | X |  | X | X |  |
| Little (2015) |  |  |  |  |  |  |  |  |  |  |  |  |  |
| Little (2019) |  |  |  |  |  |  |  |  |  |  |  |  |  |
| Berg (2005) | X |  |  |  |  |  |  |  | X |  |  |  |  |

*Note. “X” denotes that the factor identified as relevant. HRDD indicates the paper examined the specific occurrence of Housing Related Delayed Discharge but did not comment on the specific accommodation need.*

FACTORS ASSOCIATED WITH DELAY

Ten studies in this review examined the factors associated with delayed discharge. In this review, the authors separated such factors into demographic and clinical factors, as per below. Table 2 displays the demographic factors examined in each study and Table 3 the clinical factors.

***Demographic Factors***

*1. Gender:* Eleven studies in total commented on gender, and six studies conducted significance testing examining associations of delayed discharge with gender. Only two found a significant relationship, with both studies examining a Canadian sample [14, 15]. The first found a higher proportion of men in the delayed discharge group, compared to a non-delayed group (p<0.001) [14]. The second study found that higher proportion of men to women were delayed (p<0.001) [15]. This suggests in Canada, gender might be a factor influencing delays, though the same cannot be said for other countries. In studies where significance testing was not conducted, there was varying results regarding the proportions of males and females in the delayed groups.

*2. Age:* Age of patients experiencing delayed discharge was examined (n=10) with six studies exploring significant associations. Three studies identified a significant association [6, 14, 15]. Two of these were in Canadian samples and both identified significantly more adults over 44 in the delayed discharge group than younger adults [14, 15]. However, in the delayed discharged group in an English sample, younger people experienced significantly longer stays than older people [6]. No significance was found in the other three studies significantly examining age. One further study reported that the delayed group had a higher mean age [11] and another found that levels of delay was highest for older people with mental health difficulties compared to other groups [10].

*3. Ethnicity:* Of the studies that looked at ethnicity (n=5), only one found a significant association. It was found that in an English PICU Black ethnicity was associated with prolonged delay (p=0.032) compared to White ethnicity [16]. In another study looking across English psychiatric settings, it was similarly commented that Black Caribbean patients were overrepresented in delayed discharges, though significance was not tested in this study [6]. Two studies did not find a significant association with ethnicity [12, 17]. The final paper that explored ethnicity/nationality found that the majority of their sample had a country of birth of Australia, where the study was based compared to other countries of birth [8].

*4. Language spoken:* Three studies looked at language spoken. Two Canadian studies found that significantly more people in the delayed group spoke a different language [14, 15]. One Australian study however, did not find a significant association with primary language spoken and delays [12]

*5. Employment status:* Three studies looked at the relevance of employment status of patients on delays. Two of these were Australian studies, though only one examined significance. It was found that being employed significantly reduced likelihood of HRDD, with only one of the HRDD sample employed at admission [12]. In the second sample, 92% of HRDD patients were unemployed at admission [8]. One English study examined employment status, similarly, finding that only one patient with delayed discharge was employed prior to admission [6]. Significance testing was not conducted in this study.

*6. Source of income:* Only two studies looked specifically at source of income, both Australian. The first found no significant association with source of income or NDIS status with delayed discharge [12]. The second study found that the majority of their delayed sample were on disability support pensions, however significance testing was not conducted to explore associations [8].

*7. Marital status:* Five studies examined marital status. In one Australian study, not having a partner significantly predicted delays [12]. In another Australian sample, the majority (93%) of a HRDD sample were not married [8], perhaps suggesting a link. Similarly, in a Canadian sample, there was significantly less of the delayed sample married compared to the non-delayed group [14]. This significance was not supported in another Canadian sample [15]. In one English delayed discharge sample, there was no significant relationship in marital status [17].

*8. Living situation:* Living situation was examined in six studies. Living circumstances at admission was significantly associated to delayed discharge in two studies. One Canadian study found that a significantly higher proportion of delayed discharge patients were homeless compared to those not delayed [14]. In an Australian sample, the HRDD group had significantly less stable housing on admission and discharge compared to the non-HRDD group, with more of the delayed group having precarious/homeless housing status [12]. A study in Australia reported that the majority of their delayed sample were housed, though 36% were homeless [8]. This was not compared to a non-delayed group. One Canadian sample [14] and two English studies [5, 17] found no significant relationship with living situation. There was also no association found with living more than 100miles from the inpatient setting and delays in and English PICU study [7].

*9. Social support:* Four studies examined social support in relation to delayed discharge. One study identified that being visited less by a social relation was significantly associated with delayed discharge and more of the delayed group experienced social isolation [15]. Though, severe symptoms related to social withdrawal had lower odds of delayed status [15]. The other studies did not identify any significant associations. A study in Australia however did find that staff thought lack of support network contributed to delayed discharge [8].

|  | **Gender** | **Age** | **Ethnicity** | **Language** | **Employment** | | **Income** | | **Marital status** | **Living situation** | **Social Support** |
| --- | --- | --- | --- | --- | --- | --- | --- | --- | --- | --- | --- |
| **Onyon (2006)** |  |  | **🗸*** |  | |  | |  |  |  |  |
| **Haw (2017)** | **🗸** | **🗸** |  |  | |  | |  |  | **🗸** |  |
| **Tyrer (2006)** | **🗸** | **🗸** | **🗸** |  | |  | |  | **🗸** | **🗸** |  |
| **Commander (2016)** |  |  |  |  | |  | |  |  |  |  |
| **Impey (2013)** | X | X |  |  | |  | |  |  |  |  |
| **Cowman (2016)** |  |  |  |  | |  | |  |  |  |  |
| **Lewis (2006)** |  | X |  |  | |  | |  |  |  |  |
| **Paton (2004)** |  |  |  |  | |  | |  |  |  |  |
| **Hanif (2008)** |  |  |  |  | |  | |  |  |  |  |
| **Tucker (2017)** | **🗸** |  |  |  | |  | |  |  | **🗸** |  |
| **Poole (2014)** | X | **🗸*** | X |  | | X | |  |  |  |  |
| **Chuah (2022)** | X | X |  |  | |  | |  |  |  |  |
| **Honey (2022)** | **🗸** | **🗸** | **🗸** | **🗸** | | **🗸*** | | **🗸** | **🗸*** | **🗸*** | **🗸** |
| **Nguyen (2022)** | X |  | X |  | | X | | X | X | X | X |
| **Aflalo (2015)** |  |  |  |  | |  | |  |  |  | **🗸** |
| **Little (2015)** | **🗸*** | **🗸*** |  | **🗸*** | |  | |  | **🗸*** | **🗸*** |  |
| **Little (2019)** | **🗸*** | **🗸*** |  | **🗸*** | |  | |  | **🗸** |  | **🗸*** |
| **Berg (2005)** | X | X |  |  | |  | |  |  |  |  |

**Table 2** Table depicting which studies examined demographic variables

*Note.* A symbol of ‘X’ indicates that this variable was examined in the paper. A tick symbol ‘**🗸’** indicates that significance testing was conducted. A symbol of ‘*****” indicates significant finding.

***Clinical Factors***

*1. Mental Health Diagnosis:* A diagnosis of schizophrenia or psychotic disorder was significantly associated with delayed discharge, in one Canadian study [14], two English studies [7, 16] and one Australian study [12]. In a further five studies most of the delayed sample had schizophrenia diagnosis [8, 9, 11, 18]. One study also identified that all delayed patients had a psychotic disorder diagnosis, but this was not true for the non-delayed group [13]. For example, 73% in one Australian HRDD sample [8] and 42% in a Canadian delayed sample [9]. However, schizophrenia diagnosis was not significantly associated with delays in one study [15]. In two Canadian studies, mood disorders were significantly less present in delayed group [14, 15]. Personality disorder was not significantly associated with delayed discharge in one study [7]. Number of mental health diagnoses also was not significantly associated with delays in two studies [9, 12].

*2. Cognitive Disorders/Dementia:* Cognitive disorders and impairment were significantly related to delays in two Canadian studies [14, 15] and one English study [5]. Another English study, in older adult samples, identified that most (19/27) of their delayed group had diagnosis of dementia [3]. However, this study did not compare to a non-delayed group. In one Australian sample, five of the HRDD group had dementia or cognitive disorder, but none of the non-delayed group, though significance testing was not conducted on this variable due to the low numbers of people with this presentation [12]. Staff in another Australian sample commented that they thought that dementia/cognitive impairment contributed to HRDD [8].

*3. Neurodevelopmental Disorders/Learning Disabilities:* Five of the included studies commented on patients’ diagnosis of neurodevelopmental disorders, including ADHD and Autism, or Learning Disabilities. No significant association was found between neurodevelopmental disorders and delays, despite occurrence being higher in the HRDD group in an Australian sample [12]. Clinicians in another Australian study suggested that neurodevelopmental disorders contributed to delays [8]. In a Canadian sample, similarly intellectual disabilities were significantly higher in the delayed group [15]. One English study described that the LD group had complex needs, requiring additional support, which impacted delays [10]. In addition, one study found significant associations of delays with disorders of childhood/adolescence [15].

*4. Physical Health Comorbidities:* Being in fair-excellent physical health significantly predicted delays in one English study conducted in older adult inpatient settings [5]. However physical health comorbidity was significantly associated with higher odds of HRDD in an Australian sample [12]. Another Australian study qualitatively found that staff thought physical illness and disability contributed to delay and this was identified as relevant in 25% of the delayed patients records [8].

*5. Risk:* Two Australian papers looked at relevance of risk. Client experiences of self-harm was significantly related to HRDD, in one study [12]. History of violence or aggressive behaviour was also significantly associated with delays [12]. This was supported by staff qualitative reports who stated that aggressive behaviour, suicidal attempt/ideation and self-harm contributed to delays [8]. A record review in this study found risk to be a factor in 29% of delayed cases [8]. One Canadian study also found that scoring higher on the Aggressive Behaviour Scale significantly associated with delays [15].

*6. Substance use:* Substance use was not found to significantly associate with delayed discharge in an Australian study [12], though another Australian study found that clinicians qualitatively reported that drug and alcohol use contributed to delays, and this was relevant in 56% of delayed cases [8]. In one English delayed sample, 100% used substances [6], though significance testing was not conducted. However, in two Canadian studies it was found there were significantly less people experiencing substance use disorders in the delayed group [14] and significantly less people identified as struggling with past or current substance use [15].

*7. Forensic factors:* Only two studies assessed relevance of forensic history in delayed discharge, both Australian HRDD studies. One study found that being involved with the justice system reduced likelihood of HRDD [12], though not significantly. However, a history of aggressive behaviour and a history of criminal behaviour both significantly predicted delay in this study [12]. Another study highlighted that 63% of people with HRDD had a history of violent behaviour and clinicians reported they thought that history of violence, property damage and current police charges contributed to delays [8].

*8. Contact with services:* Five studies examined patients service involvement. One Canadian study found those delayed received significantly more days of contact with professionals in the week preceding admission, than non-delayed patients [14]. Another Canadian study identified that having six or more previous admissions, significantly increased odds of delay [15]. Similarly, an English study identified that those receiving formal social care before admission were significantly more likely to experience delayed discharge [5]. Though, this study did not find a significant association with mental health support pre-admission. In another study a high proportion of the delayed discharge group had been in contact with services for over five years [6]. Furthermore, most younger delayed patients (78.5%) had been previously admitted, but the same was not found for the older delayed group [6]. One final study found that there were a slightly higher mean number of earlier referrals in the delayed group, though significance testing has not been conducted to further explore this link [13].

*9. Admission factors:* Six studies examined admission factors and delays, with three identifying a link. In an English PICU, people admitted from other inpatient units or from the community were more likely to experience delay than those admitted from police stations or prisons [16]. However, in an acute psychiatric sample there was no significant relationship between form of admission and delays [17]. In an older adult sample, reason for admission was not found to be significantly associated with delays [5]. An Australian sample found that legal status during admission was significantly associated with delays, with more of the delayed group admitted under involuntary status [12]. An English study also commented on section detained under, finding most of the younger delayed group were detained under a Mental Health Act section, but less of the older adult delayed group [6]. Associations in this study were not further explored. The study conducted in Norway also commented on section at admission, but no clear link to delays was identified [13].

*10. Discharge factors:* Four studies examined discharge factors. In an English PICU, discharge to acute ward was significantly associated with delays [7]. In an English older adult sample, individuals discharged to what was described as ‘Elderly Mentally Infirm’ unit or nursing home were impacted with delays the most, though significant testing to explore this association was not conducted [3]. In an Australian study, NDIS active participant or applying for NDIS was significantly associated with delay, though there was no significant association with discharge order [12]. One other study in Australia, reported that intended discharge destination varied in their delayed sample, but links between discharge destination and delay was not explored [18].

*11. Insight:* Two studies commented on insight. Clinicians in an Australian sample qualitatively reported lack of insight contributed to HRDD [8]. One Canadian sample found a significantly higher proportion of people with limited to no insight in their delayed group [15].

*12. Other:* There were a variety of other characteristics which were only examined in one study. One Australian study found that being a victim of violence was not associated with delays [12]. In another study, it was found that clinicians thought that squalor issues, guardianship, community treatment order and financial management orders contributed to delays [8]. Finally, a Canadian study found that impaired activities of daily living was associated with delays [15]. It might be of benefit to further examine these factors in future studies.

**Table 3** Table depicting which studies examined clinical variables

|  | **Mental Health Diagnosis** | | **Cognitive Disorders** | **Neuro-development disorders** | **Physical Health** | **Risk** | **Substance Use** | **Forensic factors** | **Service contact** | **Admission factors** | **Discharge factors** | **Insight** |
| --- | --- | --- | --- | --- | --- | --- | --- | --- | --- | --- | --- | --- |
| **Onyon (2006)** | | **🗸*** |  |  |  |  |  |  |  | **🗸*** |  |  |
| **Haw (2017)** | | **🗸*** |  |  |  |  |  |  |  |  | **🗸*** |  |
| **Tyrer (2006)** | |  |  |  |  |  |  |  |  | **🗸** |  |  |
| **Commander (2016)** | |  |  |  |  |  |  |  |  |  |  |  |
| **Impey (2013)** | | X |  |  |  |  |  |  |  |  |  |  |
| **Cowman (2016)** | |  |  |  |  |  |  |  |  |  |  |  |
| **Lewis (2006)** | |  |  | X |  |  |  |  |  |  |  |  |
| **Paton (2004)** | |  |  |  |  |  |  |  |  |  |  |  |
| **Hanif (2008)** | | X | X |  |  |  |  |  |  |  | X |  |
| **Tucker (2017)** | |  | **🗸*** |  | **🗸*** |  |  |  | **🗸*** | **🗸** |  |  |
| **Poole (2014)** | |  |  |  |  |  | X |  | X | X |  |  |
| **Chuah (2022)** | | X |  |  | X |  |  |  |  |  | X |  |
| **Honey (2022)** | | **🗸*** | X | **🗸** | **🗸*** | **🗸*** | **🗸** | **🗸*** |  | **🗸*** | **🗸*** | X |
| **Nguyen (2022)** | | X | X | X | X | X | X | X |  |  |  |  |
| **Aflalo (2015)** | | **🗸** |  |  |  |  |  |  |  |  |  |  |
| **Little (2015)** | | **🗸*** | **🗸*** |  |  |  | **🗸*** |  | **🗸*** |  |  |  |
| **Little (2019)** | | **🗸** | **🗸*** | **🗸*** |  | **🗸*** | **🗸*** |  | **🗸*** |  |  | **🗸*** |
| **Berg (2005)** | | X |  |  |  |  |  |  | X | X |  |  |

*Note.* A symbol of ‘X’ indicates that this variable was examined in the paper. A tick symbol ‘**🗸’** indicates that significance testing was conducted. A symbol of ‘*****” indicates significant finding.

***Other Findings: Prevalence of Delayed Discharge***

Fourteen studies examined the prevalence of delayed discharge in psychiatric settings. However, there was variation in the way prevalence was measured.

Thirteen studies commented on the number of patients who experienced delays. Across all studies, the overall range of patients delayed was 3.5% [8, 14] to 56.9% [5]. The prevalence was highest in England, with studies in this country reporting 18% [11] to 56.9% [5] of patients experiencing a delay. This is based on the nine studies that examined prevalence in English settings. Two studies reported prevalence in Canadian psychiatric units, finding 3.5% [14] and 19% [9] of inpatients experienced delay, much lower than in England. In the study conducted in Norway [13], 9.5% to 39.1% of the sample experienced delay. In an Australian sample, 3.5% of people experienced HRDD [8]. The Australian sample represented one of the lowest prevalence of delayed patients. Perhaps this lower proportion is related to the specific type of delay measured.

Five of the included studies evaluated prevalence by calculating the number of delayed days. This range varied from 5.7% [14] to 54.8% [13] of total inpatient days. This highest percentage was in Norway adult psychiatric care [13], while the lowest number of days delayed was found in Canada [14]. In an Australian sample, 11.6% of days were attributed to HRDD [8]. Within England, the number of delayed days ranged 7% [10] to 23.5% [3] in two studies.

**References**

1. Cowman J, Whitty P. Prevalence of housing needs among inpatients: a 1 year audit of housing needs in the acute mental health unit in Tallaght Hospital. Irish Journal of Psychological Medicine. 2016;33(3):159-64.

2. Commander M, Rooprai D. Survey of long-stay patients on acute psychiatric wards. Psychiatric Bulletin. 2008;32(10):380-3.

3. Hanif I, Rathod B. Delays in discharging elderly psychiatric in-patients. Psychiatric Bulletin. 2008;32(6):211-3.

4. Paton J, Fahy M, Livingston G. Delayed discharge—a solvable problem? The place of intermediate care in mental health care of older people. Aging & mental health. 2004;8(1):34-9.

5. Tucker S, Hargreaves C, Wilberforce M, Brand C, Challis D. What becomes of people admitted to acute old age psychiatry wards? An exploration of factors affecting length of stay, delayed discharge and discharge destination. International journal of geriatric psychiatry. 2017;32(9):1027-36.

6. Poole R, Pearsall A, Ryan T. Delayed discharges in an urban in-patient mental health service in England. The Psychiatric Bulletin. 2014;38(2):66-70.

7. Haw C, Otuwehinmi O, Kotterbova E. Out of area admissions to two independent sector PICUs: patient characteristics, length of stay and delayed discharges. Journal of Psychiatric Intensive Care. 2017;13(1):27-36.

8. Nguyen J, Honey A, Arblaster K, Heard R. Housing‐related delayed discharge from inpatient mental health units: Magnitude and contributors in a metropolitan mental health service. Australian Journal of Social Issues. 2022;57(1):144-63.

9. Aflalo M, Soucy N, Xue X, Colacone A, Jourdenais E, Boivin J-F. Characteristics and needs of psychiatric patients with prolonged hospital stay. The Canadian Journal of Psychiatry. 2015;60(4):181-8.

10. Lewis R, Glasby J. Delayed discharge from mental health hospitals: results of an English postal survey. Health & social care in the community. 2006;14(3):225-30.

11. Impey M, Milner E. Delayed discharge from mental health inpatient care in the UK. Mental health practice. 2013;16(9).

12. Honey A, Arblaster K, Nguyen J, Heard R. Predicting Housing Related Delayed Discharge from Mental Health Inpatient Units: A Case Control Study. Administration and Policy in Mental Health and Mental Health Services Research. 2022;49(6):962-72.

13. Berg JE, Restan A. Duration of bed occupancy as calculated at a random chosen day in an acute care ward. Implications for the use of scarce resources in psychiatric care. Annals of General Psychiatry. 2005;4(1):1-6.

14. Little J, Hirdes JP, Daniel I, editors. ALC status in in-patient mental health settings: Evidence based on the Ontario Mental Health Reporting System. Healthcare Management Forum; 2015: SAGE Publications Sage CA: Los Angeles, CA.

15. Little J, Hirdes JP, Perlman CM, Meyer SB. Clinical predictors of delayed discharges in inpatient mental health settings across Ontario. Administration and Policy in Mental Health and Mental Health Services Research. 2019;46:105-14.

16. Onyon R, Khan S, George M. Delayed discharges from a psychiatric intensive care unit–are we detaining patients unlawfully? Journal of Psychiatric Intensive Care. 2006;2(2):59-64.

17. Tyrer P, Suryanarayan G, Rao B, Cicchetti D, Fulop N, Roberts F, et al. The bed requirement inventory: a simple measure to estimate the need for a psychiatric bed. International journal of social psychiatry. 2006;52(3):267-77.

18. Chuah CPT, Honey A, Arblaster K. ‘I'm institutionalised… there's not much I can do’: Lived experience of housing related delayed discharge. Australian Occupational Therapy Journal. 2022;69(5):574-84.

**Appendix B**

*Search terms for database searches*

Searches run: 15^th^ January 2022 and 5^th^ August 2022

Pubmed:

((delayed discharge*[Title/Abstract]) OR (delay discharge*[Title/Abstract]) OR (bed block*[Title/Abstract]) OR (discharge delay*[Title/Abstract]) OR (length of admission*[Title/Abstract]) OR (prolonged stay*[Title/Abstract]) OR (excess day*[Title/Abstract]) OR (length of stay*[Title/Abstract]) OR (long stay*[Title/Abstract])) AND (psychiat*[Title/Abstract]) OR (mental health*[Title/Abstract])) Filters: from 2002-2022

Embase:

('delayed discharg*':ab,ti,kw OR 'delay discharge*':ab,ti,kw OR 'bed block*':ab,ti,kw OR 'discharge delay*':ab,ti,kw OR 'length of admission*':ab,ti,kw OR 'prolonged stay*':ab,ti,kw OR 'excess day*':ab,ti,kw OR 'length of stay*':ab,ti,kw OR 'long stay*':ab,ti,kw) AND (psychiat*:ab,ti,kw OR 'mental health':ab,ti,kw) AND ([embase]/lim OR [embase classic]/lim) AND [2002-2022]/py

PsycInfo:

((title: (psychiat*) *OR* title: ("mental health")) *OR* (abstract: (psychiat*) *OR* abstract: ("mental health"))) *OR* (KEYWORDS: (psychiat*) *OR* KEYWORDS: ("mental health"))) *AND* ((title: ("delayed discharg*") *OR* title: ("delay discharg*") *OR* title: ("bed block*") *OR* title: ("discharge delay*") *OR* title: ("length of admission*") *OR* title: ("prolonged stay*") *OR* title: ("excess day*") *OR* title: ("length of stay*") *OR* title: ("long stay*") *OR* (abstract: ("delayed discharg*") *OR* abstract: ("delay discharg*") *OR* abstract: ("bed block*") *OR* abstract: ("discharge delay*") *OR* abstract: ("length of admission*") *OR* abstract: ("prolonged stay*") *OR* abstract: ("excess day*") *OR* abstract: ("length of stay*") *OR* abstract: ("long stay*") *OR* (KEYWORDS: ("delayed discharg*") *OR* KEYWORDS: ("delay discharg*") *OR* KEYWORDS: ("bed block*") *OR* KEYWORDS: ("discharge delay*") *OR* KEYWORDS: ("length of admission*") *OR* KEYWORDS: ("prolonged stay*") *OR* KEYWORDS: ("excess day*") *OR* KEYWORDS: ("length of stay*") *OR* KEYWORDS: ("long stay*"))) *AND* Year: 2002 *To* 2022

**Appendix C**

List of the high-income countries (as defined by the World Bank) that met inclusion:

| 1. Aruba |
| --- |
| 1. Andorra |
| 1. United Arab Emirates |
| 1. Antigua and Barbuda |
| 1. Australia |
| 1. Austria |
| 1. Belgium |
| 1. Bahrain |
| 1. Bahamas, The |
| 1. Bermuda |
| 1. Barbados |
| 1. Brunei Darussalam |
| 1. Canada |
| 1. Switzerland |
| 1. Channel Islands |
| 1. Chile |
| 1. Curaçao |
| 1. Cayman Islands |
| 1. Cyprus |
| 1. Czech Republic |
| 1. Germany |
| 1. Denmark |
| 1. Spain |
| 1. Estonia |
| 1. Finland |
| 1. France |
| 1. Faroe Islands |
| 1. United Kingdom |
| 1. Gibraltar |
| 1. Greece |
| 1. Greenland |
| 1. Guam |
| 1. Hong Kong SAR, China |
| 1. Croatia |
| 1. Hungary |
| 1. Isle of Man |
| 1. Ireland |
| 1. Iceland |
| 1. Israel |
| 1. Italy |
| 1. Japan |
| 1. St. Kitts and Nevis |
| 1. Korea, Rep. |
| 1. Kuwait |
| 1. Liechtenstein |
| 1. Lithuania |
| 1. Luxembourg |
| 1. Latvia |
| 1. Macao SAR, China |
| 1. St. Martin (French part) |
| 1. Monaco |
| 1. Malta |
| 1. Northern Mariana Islands |
| 1. New Caledonia |
| 1. Netherlands |
| 1. Norway |
| 1. Nauru |
| 1. New Zealand |
| 1. Oman |
| 1. Palau |
| 1. Poland |
| 1. Puerto Rico |
| 1. Portugal |
| 1. French Polynesia |
| 1. Qatar |
| 1. Saudi Arabia |
| 1. Singapore |
| 1. San Marino |
| 1. Slovak Republic |
| 1. Slovenia |
| 1. Sweden |
| 1. Sint Maarten (Dutch part) |
| 1. Seychelles |
| 1. Turks and Caicos Islands |
| 1. Trinidad and Tobago |
| 1. Taiwan, China |
| 1. Uruguay |
| 1. United States |
| 1. British Virgin Islands |
| 1. Virgin Islands (U.S.) |

World Bank. World Bank Country and Lending Groups 2022 [Available from: <https://datahelpdesk.worldbank.org/knowledgebase/articles/906519>.]

**Appendix D**

MMAT Quality Assessment Criteria

*Initial Screening Questions*

S1. Are there clear research questions?
S2. Do the collected data allow to address the research questions?

*Section 1 – Qualitative Criteria*

1.1. Is the qualitative approach appropriate to answer the research question?
1.2. Are the qualitative data collection methods adequate to address the research questions?
1.3. Are the findings adequately derived from the data?
1.4. Is the interpretation of results sufficiently substantiated by data?
1.5. Is there coherence between qualitative data sources, collection, analysis and interpretation?

*Section 4 – Quantitative Descriptive*

4.1. Is the sampling strategy relevant to address the research question?
4.2. Is the sample representative of the target population?
4.3. Are the measurements appropriate?
4.4. Is the risk of non-response bias low?
4.5. Is the statistical analysis appropriate to answer the research question?

*Section 5 – Mixed Methods*

5.1. Is there an adequate rationale for using a mixed methods design to address the research questions?
5.2. Are the different components of the study effectively integrated to answer the research question?
5.3. Are the outputs of the integration of qualitative and quantitative adequately interpreted?
5.4. Are divergences and inconsistencies between quantitative and qualitative results adequately addressed?
5.5. Do the different components of the study adhere to the quality criteria of each tradition of the methods involved?
